# Interstitial Lung Disease in Primary Biliary Cholangitis: A Cohort Prospective Study

**DOI:** 10.3390/life13020416

**Published:** 2023-02-02

**Authors:** Michail Kalashnikov, Larisa Akulkina, Michail Brovko, Viktoria Sholomova, Alisa Yanakaeva, Dzhamal Abdurakhmanov, Sergey Moiseev

**Affiliations:** 1 Tareev Clinic of Internal Diseases, Sechenov First Moscow State Medical University, 11 Rossolimo Str., Build. 5, Moscow 119435, Russia; 2 Hepatology Department, Vladimirsky Moscow Regional Research and Clinical Institute, 61/2 Shchepkina Str., Moscow 129110, Russia; 3 Faculty of Fundamental Medicine, Lomonosov Moscow State University, 27 Lomonosovsky Avenue, Build. 1, Moscow 119991, Russia

**Keywords:** interstitial lung disease, primary biliary cholangitis, sarcoid-like pattern, liver transplant-free survival

## Abstract

Interstitial lung disease (ILD) has been recognized as an extrahepatic manifestation ofprimary biliary cholangitis (PBC), althoughlimited data are available on its prevalence and clinical significance. Therefore, we evaluated the occurrence and clinical features of ILD in a cohort of PBC patients. Ninety-three individuals without concomitant rheumatic diseases were enrolled in our prospective cohort study. All patients underwent chest high-resolution computed tomography (HRCT). Liver-related and lung-related survival wereassessed. A lung-related outcome was defined as death from ILD complications; a liver-related outcome was defined as liver transplantation or death from liver cirrhosis complications. HRCT findings suggestive ofILD were detected in 38 patients (40.9%). A sarcoid-like pattern of PBC-associated ILD was the most frequent, followed by subclinical ILD and organizing pneumonia. Patients with ILD were less likely to have liver cirrhosis and liver-related symptoms and presented with higher serum immunoglobulin M(IgM) and M2 subtype antimitochondrial antibodies (AMA-M2) positivity rates. In a multivariate analysis, the absence of liver disease symptoms at the disease presentation (OR 11.509; 95% CI 1.210–109.421; *p* = 0.033), the presence of hepatic non-necrotizing epithelioid cell granulomas (OR 17.754; 95% CI 1.805–174.631; *p* = 0.014), higher serum IgM (OR 1.535; 95% CI 1.067–2.208; *p* = 0.020) and higher blood leukocyte count (OR 2.356; 95% CI 1.170–4.747; *p* = 0.016) were independent risk factors associated with ILD in PBC. More than a third of patients with ILD showed no respiratory symptoms, and only one ILD-related death occurred during a follow-up of 29.0 months (IQR 11.5; 38.0). Patients with ILD had better liver transplant-free survival.ILD in PBC had a benign course and was associated with a lower liver disease severity. PBC-associated ILD should be included in a list of differential diagnoses of ILD.

## 1. Introduction

Primary biliary cholangitis (PBC), formerly known as primary biliary cirrhosis, is an autoimmune cholestatic liver disease resulting from immune-mediated destruction of small intrahepatic bile ducts and eventually progressing to liver cirrhosis. Chronic nonsuppurative destructive cholangitis is a histopathological hallmark of PBC. Poorly formed non-necrotizing epithelioid cell granulomas can also be found, particularly in the earlier stages of the disease [1]. Immunologically, PBC is characterized by the presence of circulating M2 subtype antimitochondrial antibodies (AMA-M2), antinuclear antibodies (ANAs), and elevated serum immunoglobulin M (IgM). PBC mostly affects middle-aged females and usually presents with fatigue, pruritus, cutaneous hyperpigmentation, and hepatosplenomegaly [1]. Ursodeoxycholic acid (UDCA) can be used in PBC patients to reducecholestasis, alleviate clinical symptoms, and improve disease outcomes [1,2]. Recently, fibrates and obeticholic acid were introduced as the second-line treatments for PBC [2].

PBC can lead to multiple extrahepatic manifestations, including autoimmune thyroiditis, Sjögren syndrome (SjS), chronic tubulointerstitial nephritis, and other autoimmune diseases [3,4,5]. Interstitial lung disease (ILD) has been recognized as a rare extrahepatic manifestation in PBC. Cases of organizing pneumonia (OP) [6,7], lymphoid interstitial pneumonia [8,9], non-specific interstitial pneumonia (NSIP) [10,11], usual interstitial pneumonia (UIP) [12], pulmonary sarcoid-like granulomatosis [13] were reported in patients with PBC. Subclinical alveolitis [14,15] and pulmonary functional abnormalities [16] were also reported in PBC patients without evidence of lung involvement. It has been suggested that airway involvement and ILD in PBC may be due to concomitant Sjögren syndrome [17,18]. However, other authors did notsupport this hypothesis [19,20].

Limited data are available regarding the prevalence and clinical manifestations of ILD and its relation to specific ANAs in PBC. The objective of our single-center study was to evaluate the occurrence and clinical features of ILD in a cohort of patients with PBC.

## 2. Materials and Methods

### 2.1. Study Population and Design

In a prospective single-centercohort study, we enrolled consecutive patients with PBC who were admitted to our clinic between January 2018 and January 2022. The study was conducted in accordance with the principles of the Declaration of Helsinki and was approved by the local ethics committee of Sechenov University. All patients signed an informed consent form prior toenrollment.

PBC was diagnosed according to the criteria of the American Association for the Study of Liver Diseases [2]. Primary biliary cholangitis-autoimmune hepatitis overlap syndrome (PBC-AIH overlap) was diagnosed using Paris diagnostic criteria [21]. UDCA treatment response was assessed by Paris-II criteria [22].

Patients with concomitant autoimmune diseases such as SjS, systemic sclerosis (SSc), systemic lupus erythematosus (SLE), rheumatoid arthritis (RA), IgG4-related disease (IgG4-RD), or HCV infection were excluded from our study. Xerostomia solely or in combination with xerophthalmia was defined as sicca syndrome if the patient did not meet the classification criteria for SjS. 

### 2.2. Data Collection and Outcomes

All patients underwent chest high-resolution computed tomography (HRCT) and spirometry. HRCT patterns of ILD were classified according to the American Thoracic Society and European Respiratory Society guidelines [23]. Criteria for subclinical ILD include ILD extent < 5%, preserved lung function (FVC > 80%), and absence of respiratory symptoms. Bilateral hilar adenopathy with multifocal peribronchovascular nodules was classified as a sarcoid-like HRCT pattern. Forced expiratory volume in 1 s (FEV1) and forced vital capacity (FVC) were measured to evaluate pulmonary function [24]. Liver ANAs panel was assessed in 46 patients by enzyme-linked immunosorbent assay (ELISA) (EuroImmune, Germany). Liver stiffness was measured using transient elastography (Echosense, France).

The studied outcomes during follow-up included ILD-related death and liver transplantation or death from liver cirrhosis complications.

**Figure 1:**
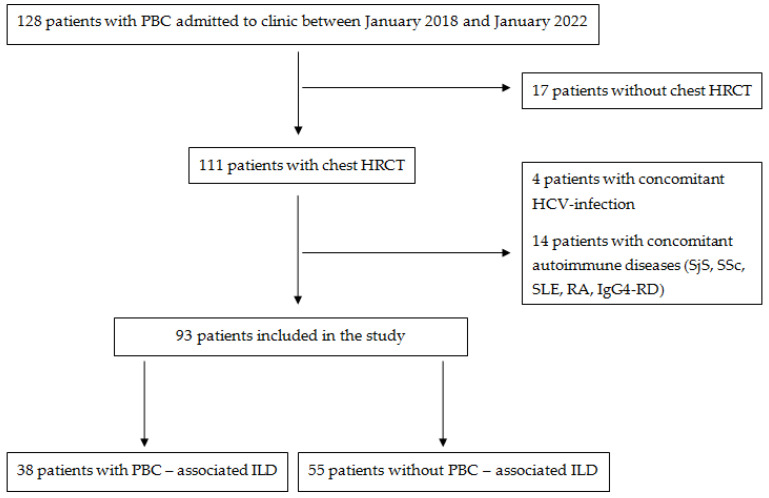
Flow diagram of the study.

The study enrollment flow is illustrated in Figure 1.

### 2.3. Statistical Analysis

The Shapiro–Wilktest was used to check if the continuous variables follow a normal distribution. Continuous variables were presented as mean with standard deviation (SD) or median with interquartile range (IQR), and categorical variables were expressed as counts and percentages. The Student’s *t*-test was used to compare normally distributed continuous variables, whereas the Mann–Whitney U test was used when data were not normally distributed. Pearson’s χ2 test and Fisher’s exact test were used for comparing frequencies. Factors associated with ILD in PBC were evaluated by logistic regression analysis. Survival analysis was performed using the Cox regression analysis, the Kaplan–Meier method, and the log-rank test. A two-sided *p*-value < 0.05 was considered statistically significant. Statistical analyses were performed using IBM SPSS version 22 (SPSS, Inc., Chicago, IL, USA).

## 3. Results

### 3.1. Patient Characteristics

A total of 93 PBC patients were enrolled and followed up for a median of 29.0 months (IQR 11.5; 38.0). Most of them were females (95.7%). The mean age at disease presentation was 47.1 ± 1.0 years. Nearly half of the patients had liver cirrhosis. Every sixth patient presented with PBC-AIH overlap syndrome. The diagnosis was confirmed by ultrasound-guided percutaneous needle liver biopsy in 45 patients. All patients were treated with UDCA, whereas patients with PBC-AIH overlap also received glucocorticoids and azathioprine. Circulating AMA-M2 wasdetected in 90.3% of the patients. VariousANAs, including anti-gp210, anti-sp100, anti-SS-A, and anti-PML, were less prevalent (Table 1).

### 3.2. Clinical Features of ILD

HRCT findings were suggestive of ILD in 38 (40.9%) of 93 PBC patients. Among them, 14 (36.8%) patients had a sarcoid-like pattern, 9 (23.7%) had subclinical ILD, 7 (18.4%) had OP, 4 (10.5%) had unclassifiable interstitial pneumonia (UnIP), 3 (7.9%) had NSIP, and 1 (2.6%) had UIP. Unclassifiable radiological patterns included multiple lung nodules in combination with diffuse ground-glass opacity in two patients, bilateral basilar interlobular septal thickening with ground-glass opacity and lung nodules in one patient, bilateral hilar lymph node enlargement with extensive ground-glass opacity and reverse halo sign in one patient.

**Figure 2:**
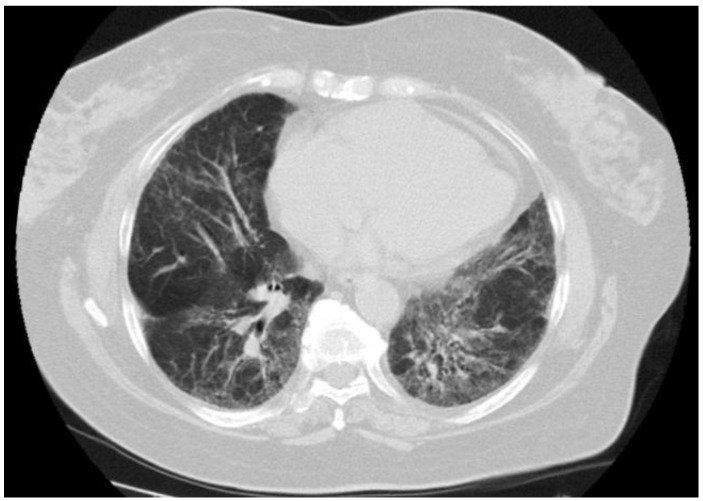
NSIP pattern in a 70-year-old female with PBC who died from progressive respiratory failure.

**Figure 3:**
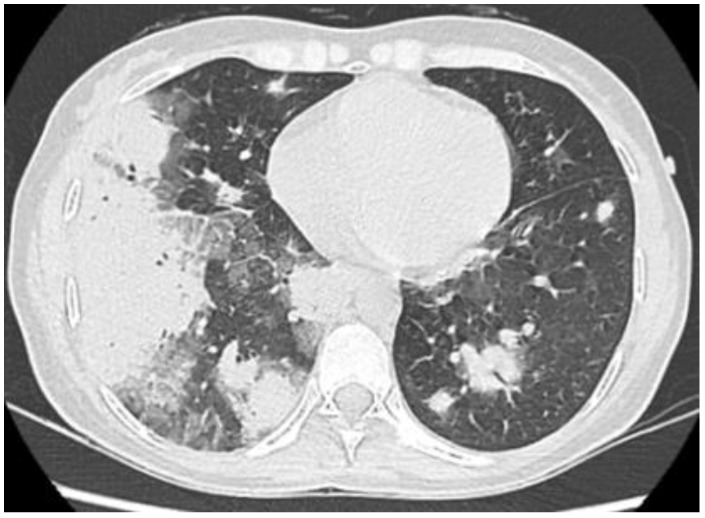
Extensive bilateral pulmonary consolidations in a 35-year-old female with PBC and OP complicated with acute respiratory failure and rapid improvement after prednisone administration.

**Figure 4:**
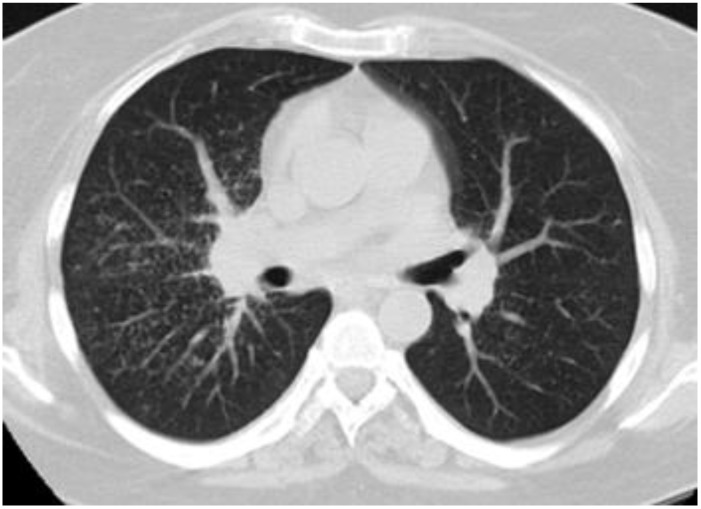
Sarcoid-like pattern in a 45-year-old female with PBC-AIH overlap.

**Figure 5:**
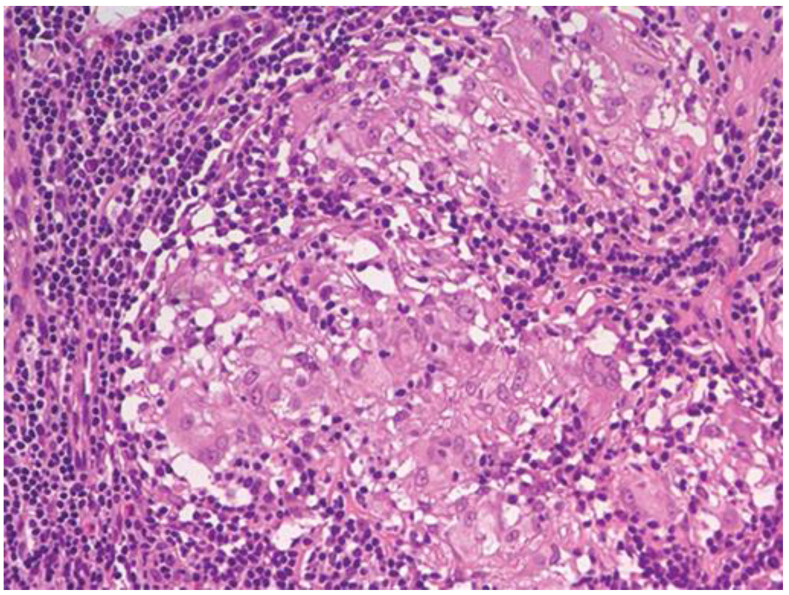
Non-necrotizing epithelioid cell granulomas in the mediastinal lymph node of a 38-year-old female with PBC and sarcoid-like HRCT pattern (hematoxylin and eosin staining, ×200).

Tissue histology was available in 9 of 14 patients with sarcoid-like patterns (lung biopsy via video-assisted thoracoscopic surgery in five, mediastinal lymph nodes in two, supraclavicular lymph nodes in two), and in all cases showed non-necrotizing epithelioid cell granulomas with giant cells. An open lung biopsy was performed on two patients with pulmonary consolidation showed granulation tissue buds in the alveoli and alveolar ductus consistent with the OP pattern. Histopathological findings in three patients with indeterminate radiological patterns included dense lymphocytic infiltration of the alveolar septa (n = 3), giant cells (n = 3), poorly formed non-necrotizing epithelioid cell granulomas (n = 2), foamy macrophages (n = 2), mild eosinophilic infiltrate (n = 2) and moderate interstitial fibrosis (n = 1). These findings were incompatible with radiological data, and we were unable to define a type of ILD. One patient with an unclassifiable radiological pattern refused a lung biopsy. Representative HRCT patterns and tissue histology are shown in Figure 2, Figure 3, Figure 4 and Figure 5.

ILD was an initial manifestation of PBC in nine (9.6%) patients. All of them had liver function tests abnormalities at the time of ILD detection. The most common clinical manifestations of ILD included chronic cough (50.0%), fever (44.7%), and dyspnea (42.1%). Respiratory failure was present in three (7.9%) of thirty-eightpatients. Fourteen (36.8%) of thirty-eightpatients with ILD had no respiratory symptoms during follow-up. 

### 3.3. Comparison of ILD and ILD-Free Patients

Patients with and without ILD were similar in age at the time of disease presentation, sex, smoking status, and the occurrence of overlap with AIH, whereas obesity was more common in patients with ILD. Liver cirrhosis was less prevalent in patients with ILD. ILD-free patients showed higher liver stiffness values. It is worth noting that patients with ILD more frequently did not present with liver disease symptoms at the disease presentation. FEV1 and FVC values did not differ significantly between the two groups (Table 1).

Hepatic non-necrotizing epithelioid cell granulomas were more common in patients with ILD compared to patients without ILD (44.4% vs. 11.1%, *p* = 0.016). Seven of eight patients with hepatic granulomas and ILD had a sarcoid-like pattern, and one patient had an OP pattern. Liver cirrhosis occurred less frequently among patients with hepatic granulomas than in patients without granulomatous liver disease (27.3% vs. 61.8%, *p* = 0.081). 

**Figure 6:**
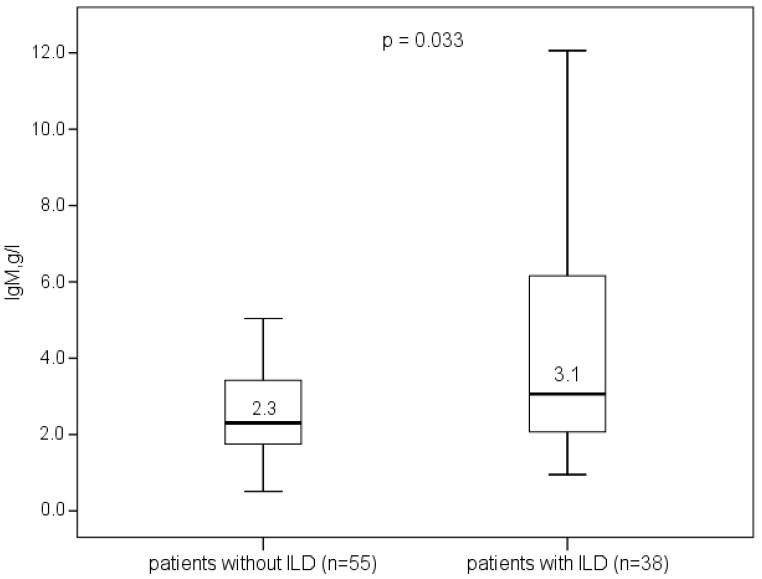
Serum IgM concentrations of patients with and without ILD.

Patients with ILD had higher blood leucocyte count, serum IgM levels (3.1 (2.1; 6.2) g/L vs. 2.3 (1.7; 3.4) g/L, *p* = 0.033, Figure 6), and the rate of AMA-M2 positivity. The percentages of patients who tested positive for anti-gp210, anti-sp100, anti-SS-A, and anti-PML were similar in the two groups. AMA-M2-positive patients had higher serum IgM levels than AMA-M2-negative patients (2.7 (1.9; 4.5) g/L vs. 1.8 (1.3; 2.7) g/L, *p* = 0.05).

### 3.4. Factors Associated with ILD in PBC

Multivariate logistic regression analysis adjusted for age, gender, and smoking status showed that the absence of liver disease symptoms at the disease presentation (odds ratio (OR) 11.509; 95% confidence interval (CI) 1.210–109.421; *p* = 0.033), the presence of hepatic non-necrotizing epithelioid cell granulomas (OR 17.754; 95% CI 1.805–174.631; *p* = 0.014), higher serum IgM (OR 1.535; 95% CI 1.067–2.208; *p* = 0.020), and higher blood leukocytecount (OR 2.356; 95% CI 1.170–4.747; *p* = 0.016) were independently associated with ILD in PBC (Table 2).

### 3.5. Follow-Up

Repeated chest HRCT was conducted in 34 patients with ILD at an interval of 3–6 months. Immunosuppressive agents were started in case of radiological and/or clinical deterioration of ILD. A total of 16 patients were treated for ILD with azathioprine or methotrexate in combination with systemic glucocorticoid. Improvement in chest HRCT was shown in 22 patients. It was spontaneous in eight patients (sarcoid-like pattern in three, subclinical ILD in three, and OP in two) or induced by immunosuppression in 14 patients (sarcoid-like pattern in eight, OP in four, UnIP in two). ILD was stable in fivepatients who received no immunosuppression (subclinical ILD in fourand sarcoid-like pattern in one) and in two patients treated with immunosuppressive agents (sarcoid-like pattern and UIP). Radiological progression of ILD was found in five patients. Two patients with NSIP and UnIP died from liver cirrhosis complications before initiation of immunosuppressive treatment, whereas two patients with OP and UnIP had no respiratory symptoms and were clinically stable. One patient showed no ILD response to immunosuppressive agents. This was a 70-year-old female with NSIP (Figure 2) who developed severe pulmonary hypertension and died from respiratory failure. The autopsy revealed extensive interstitial pulmonary fibrosis consistent with fibrosing NSIP. During follow-up, only this patient died from ILD complications.

### 3.6. Survival Analysis

**Figure 7:**
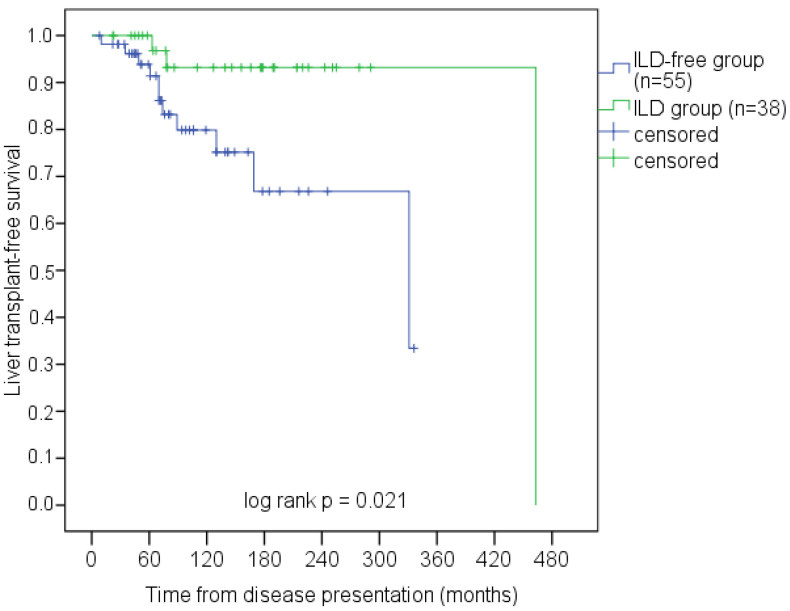
Liver-related survival of PBC patients by the ILD presence.

At the end of the study, there were 12 deaths from complications of liver cirrhosis and two liver transplantations. Three deceased patients had ILD, but postmortem examination was not available. The 10-year liver transplant-free survival in ILD patients was higher than in patients without ILD (93.2% vs. 79.9%, *p* = 0.021, Figure 7). 

In the univariate Cox regression analysis, ILD was associated with a lower risk of liver-related outcomes (hazard ratio (HR) 0.2; 95% CI: 0.044–0.911; *p* = 0.038), but this association lost statistical significance after adjusting for other covariates (Table 3).

## 4. Discussion

The results of our single-center cohort study showed that ILD is a common finding in PBC and can be detected on chest HRCT in up to 41% of patients without concomitant autoimmune diseases such as SjS, SSc, SLE, RA, and IgG4-RD. The most common patterns of ILD included sarcoid-like changes, subclinical ILD, and OP, whereas UnIP, NSIP, and UIP occurred less frequently. ILD was asymptomatic in one-third ofpatients. Of note, ILD manifesting by cough and/or dyspnea was the first presentation of the disease in a proportion of PBC patients (10%) who were referred to our department by pulmonologists due to elevated serum cholestatic enzymes. The absence of liver disease symptoms at the presentation of the disease was an independent risk factor for PBC-associated ILD. These data suggest that patients with unexplained ILD should be screened for asymptomatic PBC. 

In the previous studies, the prevalence of ILD in PBC patients was lower. In two Chinese cohorts, ILD was detected only in 15.7% of 178 patients and 10.0% of 109 patients, respectively [18,25]. In both studies, ILD was frequently associated with connective tissue diseases, e.g., SjS. The reported prevalence of ILD in Caucasian PBC patients was even lower and varied from 2.2% to 5.4% [26,27,28]. It was probably underreported since a plain chest roentgenogram was used to detect lung abnormalities in these studies. Of note, all aforementioned studies included a proportion of patients with concomitant connective tissue diseases (CTD) who were excluded from our cohort. A higher prevalence of ILD in our PBC patients was probably related to the use of HRCT for ILD detection, including subclinical ILD.

Sarcoid-like lung involvement was the main pattern of ILD in our study. In our opinion, PBC-associated pulmonary granulomatosis should be considered as the extrahepatic manifestation of PBC rather than as a concomitant sarcoidosis. PBC and sarcoidosis share common features, and differential diagnosis may be challenging in some cases. Both hepatic and extrahepatic non-necrotizing granulomas can be found in PBC [29], particularly in the earlier stages of the disease [30,31]. In a previous necropsy series of 120 PBC patients, intrathoracic granulomas were detected less frequently than in our study (1.6% vs. 15.0%) [28]. The bronchoalveolar lavage CD4^+^/CD8^+^lymphocytes ratio in PBC patients without lung involvement was similar to that in pulmonary sarcoidosis [15]. Hepatic granulomas in PBC, in contrast to granulomas in hepatic sarcoidosis, are poorly formed, not confluent, and localize mostly within portal tracts [32]. Ductopenia is rarely seen in hepatic sarcoidosis [32,33]. As a rule, patients with genuine hepatic sarcoidosis do not have serum AMA-M2 and respond to immunosuppressing agents [34]. Lee et al. showed a better prognosis in PBC patients having hepatic granulomas [35]. You et al. suggested that hepatic granulomas in PBC may be an adaptive reaction to chronic bile duct injury and may reduce cholangitis activity [31]. In our study, liver cirrhosis was less common among patients with hepatic granulomas, and two-thirds of these patients presented witha sarcoid-like pattern on chest HRCT. Only one of 14 patients with a sarcoid-like type of ILD died from liver cirrhosis complications. 

We identified subclinical ILD in nine (9.7%) of 93 PBC patients. Subclinical ILD was studied in patients with RA [36], SSc, antisynthetase syndrome, and mixed CTD [37] and was suggested to represent an early form of CTD-associated ILD [37,38,39]. The reported rate of radiological progression of subclinical ILD in patients with CTD varied from 34% to 57% over 1.5 to 4.5 years [37,40,41]. There was no progression of subclinical ILD during the 2.4-year follow-up in our study. Anyway, we believe that all patients with PBC should be screened with chest HRCT for subclinical ILD to improve early detection of PBC-associated ILD. 

Similar to Lee and Shen, we found non-necrotizing epithelioid cell granuloma, OP, interstitial fibrosis, and alveolar septa lymphocytic infiltration to be the predominant histopathological features of PBC-associated ILD [25,42]. It has been speculated whether these findings constitute a specific pattern of ILD in some PBC patients [42].

Every third PBC patient with ILD in our study was obese. The association between obesity and pulmonary diseases, including ILD, can be related to TNF-α -, MCP-1 -, and TGF-β1-dependent pathways, insulin resistance, or altered lung microbiota [43,44,45]. Greater amounts of visceral adipose tissue predispose to a higher prevalence of interstitial lung abnormalities [46]. It was suggested that this association mightbe partially mediated by IL-6 and leptin [46]. 

In our study, blood leukocyte and neutrophil counts were higher in patients with ILD, although they did not exceed the upper limit of the normal range. Neutrophils have been recognized as an essential player in the pathogenesis of ILD [47,48] and promote interstitial pulmonary fibrosis via several pathways [47,49]. However, higher leucocyte counts in patients with ILD could be simply due to the less prominent hypersplenism related to portal hypertension, given a lower prevalence of liver cirrhosis and a trend to a higher platelet count in these patients.

All our PBC patients with ILD tested positive for AMA-M2, which was not detected in 16.4% of ILD-free patients. Similarly, Shen et al. showed that ILD was more common in AMA-M2-positive patients than in AMA-M2-negative patients (92.3% vs. 80.0%) [25]. Liu et al. detected AMA-M2 in 10 of 11 (90.9%) PBC patients with ILD [18].

It is well known that AMA-M2 is associated with a higher serum IgM in PBC [50,51,52]. It is unclear whether elevated serum IgM is an epiphenomenon or plays a role in PBC pathogenesis. Immune complexes containing IgM were found in the affected skin and kidneys of patients with PBC [53,54,55,56]. However, it is not known whether they can induce lung injury in these patients. Immune complexes containing IgG and complement C1q, but not IgM, were also detected in the pulmonary interstitium in a patient with PBC-associated ILD [57]. Moreover, immune complexes in PBC may be formed by AMAs and AMA-antigens [58,59]. Further studies could shed light on the role of humoral immunity in PBC-associated ILD pathogenesis.

Data regarding the role of ANAs in the development of extrahepatic manifestations of PBC are scarce. Nickowitz et al. studied serum anti-gp210 and anti-Lamin B in patients with PBC and found no association with extrahepatic manifestations and associated CTD, except RA [60]. However, ILD was not mentioned in this study. Shen et al. found no significant difference in the seropositivity rates for all types of ANAs and anticardiolipin antibodies between groups of PBC patients with and without ILD [25]. Anti-gp210 was shown to be a potential predictor of adverse outcomes in PBC [61]. In our study, all PBC-specific ANAs, including anti-gp210, anti-sp100, anti-SS-A, and anti-PML, were found with similar frequency in patients with and without ILD. We suppose that these antibodies predominately target antigens of biliary epithelium and therefore are not involved in the pathogenesis of PBC-associated ILD.

The prognostic implication of ILD in PBC remains undetermined, although fatal cases were reported [62,63]. Only one of our 38 patients died from complications of progressive ILD, whereas most patients responded to glucocorticosteroids in combination with azathioprine or methotrexate and showed improvement or a stable course of lung disease. These findings suggest that ILD in PBC has a favorable prognosis if diagnosed and treated earlier.

Ten-year liver transplant-free survival in UDCA-treated PBC patients ranged from 63% to 94% [64]. Shen et al. did not find a correlation between ILD and the Mayo risk score (MRS) that is used to determine prognosis in PBC [25]. We did not assess MRS in our study. However, 10-year liver transplant-free survival in patients with ILD was higher than in patients without ILD (93.2% vs. 79.9%), although the predictive significance of lung disease was not confirmed by multivariate Cox regression analysis. Therefore, we cannot conclude that ILD was associated with better liver prognosis in PBC patients.

Our study has limitations. It was conducted in a single center, and the study sample was relatively small. The duration of follow-up was probably insufficient for adequate assessment of lung and liver-related outcomes. The number of lung-related outcomes was too small for survival analysis. Prospective multicentre cohort studies are needed to validate our results.

## 5. Conclusions

We present the results of the prospective single-center cohort study investigating the prevalence, clinical features, and risk factors for ILD in Russian patients with PBC. The diagnostic challenges of the diagnosis of asymptomatic PBC in unexplained ILD are highlighted. Apparently, PBC-associated ILD should be included in a list of differential diagnoses of ILD.

ILD was highly prevalent in our cohort (up to 41%). The most common HRCT patterns of ILD-PBC included sarcoid-like changes, followed by subclinical ILD and OP. The absence of liver disease symptoms at the disease presentation, the presence of hepatic non-necrotizing epithelioid cell granulomas, higher serum IgM, and higher blood leukocyte count were independent risk factors for ILD. Most PBC patients showed radiological improvement of ILD after immunosuppressive therapy and a favorable short-term prognosis of ILD.

This study is the first to report the prognostic implication of ILD for the liver disease course in PBC. PBC-related ILD was associated with a lower liver disease severity, although it was not an independent factor for better liver-transplant-free survival.

## Figures and Tables

**Table 1:** Characteristics of patients with and without ILD.

Parameters	All Patients (n = 93)	Patients with ILD (n = 38)	Patients without ILD (n = 55)	*p* Value
Female, *n* (%)	89 (95.7%)	37 (97.4%)	52 (94.5%)	0.642
Age of disease presentation, years, mean ± SD	47.1 ± 1.0	45.4 ± 10.4	48.2 ± 9.5	0.181
Ever-smokers, *n* (%)	18 (19.4%)	7 (18.4%)	11 (20.0%)	1.0
Obesity, *n* (%)	21 (22.6%)	13 (34.2%)	8 (14.5%)	0.042
PBC-AIH overlap, *n* (%)	16 (17.2%)	9 (23.7%)	7 (12.7%)	0.263
Liver cirrhosis, *n* (%)	49 (52.7%)	15 (39.5%)	34 (61.8%)	0.034
Hepatic granulomas, *n* (%)	11/45 (24.4%)	8/18 (44.4%)	3/27 (11.1%)	0.016
Liver stiffness, kPa, *Me* (IQR)	11,4 (6.9; 17.4)	7.9 (5.6; 12.6)	14.2 (8.2; 26.3)	0.006
FEV1, % predicted, *Me* (IQR)	102.0 (88.7; 115.7)	99.5 (90.0; 117.2)	105.0 (88.0; 110.7)	0.807
FVC, % predicted, *Me* (IQR)	101.0 (90.0; 110.0)	99 (85.0; 114.3)	103 (91.5; 110.0)	0.373
Absence of liver disease symptoms at the disease presentation, *n* (%)	33 (35.5%)	20 (52.6%)	13 (23.6%)	0.004
Pruritus, *n* (%)	63 (67.7%)	20 (52.6%)	43 (78.2%)	0.01
Cutaneus hyperpigmentation, *n* (%)	37 (39.8%)	8 (21.1%)	29 (52.7%)	0.002
Ascites, *n* (%)	16 (17.2%)	4 (10.5%)	12(21.8%)	0.176
Esophageal varices, *n* (%)	36 (38.7%)	13 (34.2%)	23 (41.8%)	0.459
Hepatic encephalopathy, *n* (%)	11 (11.8%)	2 (5.3%)	9 (16.4%)	0.190
Sicca syndrome, *n* (%)	34 (36.6%)	13 (34.2%)	21 (38.2%)	0.696
Arthralgias, *n* (%)	33 (35.5%)	17 (44.7%)	16 (29.1%)	0.121
Fever, *n* (%)	21 (22.6%)	17 (44.7%)	4 (7.3%)	<0.001
Dyspnea, *n* (%)	22 (23.7%)	16 (42.1%)	6 (10.9%)	0.001
Chronic cough, *n* (%)	25 (26.9%)	19 (50.0%)	6 (10.9%)	<0.001
Platelet count, *10^9^/L, mean ± SD	194.6 ± 9.9	215.7 ± 81.7	180.4 ± 102.5	0.083
Leucocyte count, *10^9^/L, *Me* (IQR)	5.2 (3.8; 6.7)	6.0 (4.6; 7.4)	4.4 (3.4; 6.0)	0.001
Neutrophil count, *10^9^/L, *Me* (IQR)	2.8 (2.0; 3.7)	3,2 (2.6; 4.5)	2,5 (2.0; 3.1)	0.003
Albumin, g/L, *Me* (IQR)	40.6 (36.0; 42.6)	40.3 (36.4; 42.0)	40.6 (35.3; 42.8)	0.827
Total bilirubin, μmol/L, *Me* (IQR)	21.4 (13.1; 39.5)	16.7 (12.5; 30.8)	23.2 (15.5; 46.4)	0.084
Alkaline phosphatase, U/L, *Me* (IQR)	507.0 (296.5; 1018.7)	585.0 (230.5; 1053.0)	487.0 (319.0; 945.0)	0.914
Gamma-glutamyl transferase, U/L, *Me* (IQR)	128.5 (45.0; 339.0)	148.0 (42.5; 463.0)	124.0 (48.0; 255.0)	0.602
IgM (g/L), *Me* (IQR)	2.6 (1.8; 4.2)	3.1 (2.1; 6.2)	2.3 (1.7; 3.4)	0.033
Rheumatoid factor positivity, *n* (%),	25/75 (33.3%)	13/32 (40.6%)	12/43 (27.9%)	0.248
AMA-M2 positivity, *n* (%)	84 (90.3%)	38 (100%)	46 (83.6%)	0.01
anti-gp210 positivity, *n* (%)	22/46 (47.8%)	5/14 (35.7%)	17/32 (53.1%)	0.346
anti-sp100 positivity, *n* (%)	16/46 (34.8%)	4/14 (28.6%)	12/32 (37.5%)	0.739
anti-PML positivity, *n* (%)	5/46 (10.9%)	1/14 (7.1%)	4/32 (12.5%)	1.0
anti-SS-A positivity, *n* (%)	10/52 (19.2%)	3/18 (16.7%)	7/34 (20.6%)	1.0
UDCA response, *n* (%)	49/91 (53.8%)	24/37 (64.9%)	25/54 (46.3%)	0.081

**Table 2:** Factors associated with ILD in PBC patients.

	Univariate Logistic Regression Analysis		Multivariate Logistic Regression Analysis	
	OR (95% CI)	*p* Value	OR (95% CI)	*p* Value
Age of disease presentation	0.971 (0.930–1.014)	0.181		
Female gender	2.135 (0.214–21.336)	0.519		
Ever-smokers	0.903 (0.315–2.589)	0.85		
Obesity	3.055 (1.118–8.350)	0.029		
Absence of liver disease symptoms at the disease presentation	3.590 (1.474–8.743)	0.005	11.509 (1.210–109.421)	0.033
Pruritus	0.310 (0.126–0.765)	0.011		
Cutaneus hyperpigmentation	0.239 (0.093–0.614)	0.003		
Liver cirrhosis	0.403 (0.173–0.941)	0.036		
Hepatic granulomas	6.4 (1.402–29.209)	0.017	17.754 (1.805–174.631)	0.014
Liver stiffness	0.852 (0.738–0.983)	0.028		
Leucocyte count	1.460 (1.134–1.881)	0.003	2.356 (1.170–4.747)	0.016
Platelets count	1.004 (0.999–1.009)	0.088		
Serum IgM	1.254 (1.035–1.520)	0.021	1.535 (1.067–2.208)	0.020
AMA-M2 positivity	1.825 (1.504–2.218)	0.009		
UDCA response	2.142 (0.905–5.067)	0.083		

**Table 3:** Risk factors for adverse liver-related outcome in PBC patients.

	Univariate Cox Regression Analysis		Multivariate Cox Regression Analysis	
	HR (95% CI)	*p* Value	HR (95% CI)	*p* Value
Age of disease presentation	1.038 (0.976–1.105)	0.237	1.276 (1.078–1.510)	0.005
Liver cirrhosis	57.644 (0.648–5128.411)	0.077		
Ascites	15.047 (4.087–55.399)	<0.001		
Esophageal varices	9.010 (1.985–40.893)	0.004		
Hepatic encephalopathy	25.885 (7.042–95.147)	<0.001	53.950 (2.404–1210.699)	0.012
Albumin	0.834 (0.770–0.904)	<0.001		
Total bilirubin	1.012 (1.007–1.016)	<0.001		
Prothrombin index	0.956 (0.931–0.983)	0.001		
ILD presence	0.2 (0.044–0.911)	0.038		
UDCA response	0.089 (0.018–0.437)	0.003		

## Data Availability

The datasets used during the current study areavailablefrom the corresponding author upon reasonable request.
